# Western Flora

**Published:** 1834

**Authors:** 


### V. WESTERN FLORA.

Mr. Riddell's Western Flora will be completed in the next number, so that the whole will be comprised in the same volume. Hoping that our feeble efforts, on former occasions, to awaken in the West, an attention to the study of Botany, have not been wholly unavailing, we flatter ourselves that some of our readers will be gratified with the presentation of this *prodromus* of a perfect catalogue of Western plants at the opening of spring, when the first bloom of the fields and woods cannot fail to invite them abroad. We regard Mr. Riddell as an accurate naturalist, and presume that his Flora, will furnish important facilities to the students of Western Botany.

We may mention that he has had a small edition struck off distinct from the Journal, copies of which, after the month of March, may be had of our booksellers.

